# Prognostic significance of PD-L1 in solid tumor: An updated meta-analysis

**DOI:** 10.1097/MD.0000000000006369

**Published:** 2017-05-05

**Authors:** Qianqian Wang, Fang Liu, Lei Liu

**Affiliations:** a Laboratory of Molecular Diagnosis of Cancer; b Cancer Center, State Key Laboratory of Biotherapy, West China Hospital, Sichuan University, Chengdu, Sichuan, China.

**Keywords:** meta-analysis, PD-L1, prognosis, solid tumor

## Abstract

**Background:**

An increasing number of studies have examined the ability of programmed death-ligand 1 (PD-L1) to function as a marker for tumor prognosis. However, whether PD-L1 expression is a prognostic factor for the poor outcomes in many human cancers remains controversial. This study aims to investigate the prognostic role of PD-L1 expression through a meta-analysis update of 60 studies.

**Methods:**

The studies were identified by searching PubMed, Embase, Google Scholar, and Cochrane Library, and were assessed by further quality evaluation. The pooled hazard ratios (HRs) with 95% confidence intervals (CIs) for total and stratified analyses were calculated to investigate the association between the PD-L1 expression and the overall (OS) and disease-free (DFS) or progression-free survivals (PFS) of cancer patients. Heterogeneity and publication bias were also investigated.

**Results:**

The results indicated that PD-L1 overexpression can predict a poor OS (HR = 1.58, 95% CI = 1.38–1.81, *P* <.000) and DFS/PFS (HR = 1.72, 95% CI = 1.26–2.33, *P* = .001). Subgroup analyses showed that PD-L1 overexpression was significantly related to the poor OS in patients with breast (HR = 1.98, 95% CI = 1.15–3.41, *P* = .014), urothelial (HR = 2.24, 95% CI = 1.61–3.12, *P* <.000), renal (HR = 3.30, 95% CI = 2.23–4.86, *P* <.000), and gastric cancers (HR = 1.56, 95% CI = 1.02–2.37, *P* = .040). Furthermore, PD-L1 overexpresion was significantly associated with poor DFS/PFS in patients with hepatocellular carcinoma (HCC) (HR = 1.72, 95% CI = 1.21–2.46, *P* = .003), melanoma (HR = 3.39, 95% CI = 2.02–5.69, *P* <.000), and renal carcinoma, (HR = 5.04, 95% CI = 2.87–8.86, *P* <.000). The adverse prognostic impact of PD-L1 was observed in patients of different ethnicities.

**Conclusions:**

The findings of this meta-analysis suggest the correlation of PD-L1 overexpression with worse OS in patients with solid tumors. However, the correlations differed according to tumor types.

## Abstract 2

Supplemental Digital Content is available in the text

## 1 Introduction

Programmed cell death 1 (PD-1), an immunoinhibitory receptor was first described in 1992 by Ishida et al.^[1]^ After their immunological activation, PD-1 expresses on CD4+ and cluster of differentiation (CD)8+ T cells, as well as natural killer (NK) and B cells, and monocytes.^[1]^ Although PD-1 cell surface protein expression can be discovered within 24 hours of stimulation, the functional effects of PD-1 ligation are observed within a few hours following T-cell activation.^[2]^ Tumors express antigens recognized by host T cells, although the immunologic clearance of tumors is rare. Part of this failure is attributed to the immune suppression by the tumor microenvironment. PD-1 interacts with 2 ligands, PD-L1 (PD-L1 is also called B7-H1 and CD274) and PD-L2. The PD-L1 expression on many tumors is an integrant part of this suppressive milieu and may act in cooperation with other immunosuppressive signals. PD-L1 is expressed in several tumor types, such as melanoma, glioblastoma, and cancers in the lung, kidney, head and neck, stomach, colon, pancreas, breast, cervix, and ovary. PD-L1 inhibits T-cell proliferation and adhesion, as well as cytokine production. Furthermore, it appears to regulate T-cell function in peripheral tissues.^[3]^

PD-L1-positive tumors may indicate immune active tumors that can be a response to anti-PD-1 and/or PD-L1 therapies because they are correlated with the poor prognosis of many of these malignancies, including lung adenocarcinoma, melanoma, and refractory Hodgkin's lymphoma.^[4–7]^ However, the prognostic role of PD-L1 remains unclear. Other studies have found that PD-L1 expression is correlated with both poor prognosis and no prognostic significance, making it difficult for researchers to present definitive conclusions. Thus, the PD-L1 expression in cancers and its prognostic significance are still uncertain.

In this study, we conducted a systematic review and meta-analysis to summarize the global findings in using PD-L1 for the prediction of clinical results for cancer patients.

## 2 Materials and methods

### 2.1 Search strategy

We performed a network search using PubMed, Embase, Google Scholar, and Cochrane Library for original articles that analyzed the prognostic value of PD-L1 in various cancers. We selected studies using different combinations of the following keywords: “Neoplasm,” “cancer,” “carcinoma,” “PD-L1,” “programmed cell death ligand 1,” “B4-H1,” and “CD-274.” The final search was performed on November 2015. We increased the integrity and accuracy of the search process by manually screening the reference lists for included articles to explore potential studies.

As the present meta-analysis was performed based on previous published studies,thus no ethical approval and patient consent are required.

### 2.2 Study inclusion

The studies were considered eligible if they met all of the following inclusion criteria: diagnosis of solid tumor was proven in human; the expression of PD-L1 through immunochemistry was evaluated; PD-L1 was correlated with overall survival (OS), disease-free survival (DFS), or progression-free survival (PFS); and hazard ratio (HR) and their corresponding 95% confidence intervals (CI) were estimated by sufficient data. Articles were excluded based on the following criteria: review or laboratory articles, as well as case studies or letters; articles that described the survival outcome of other indicators; articles from 1 author and studies with repeated samples of the same patients; and unpublished studies.

### 2.3 Data extraction

Two investigators (Qianqian Wang and Fang Liu) independently extracted data from each eligible study. The following information were extracted in each study: author, year of publication, country, number of patients, median age, median follow-up period, binded reading, tumor stage, PD-L1 overexpression cut-off value, prognostic outcomes of interest, analytical method, and HR with its 95% CI. Disagreements were resolved through discussion, and consensus between the 2 authors.

### 2.4 Statistical analysis

Positive or negative of PD-L1 was defined according to the cut-off values provided by the authors. The impact of PD-L1 overexpression on prognosis in solid tumors was estimated by HR and its 95% CI. We directly used crude values when HRs were reported in the original studies. Otherwise, the values were calculated using Kaplan–Meier curves according to the methods described by Parmar,^[8]^ Williamson et al,^[9]^ and Tierney et al.^[10]^ Statistical heterogeneity between studies was quantified using Cochran's *Q* test and Higgins I-squared statistic. Heterogeneity was defined as *P* <.05 or *I*^2^ >50%. If there was statistically significant heterogeneity, a random effects model was selected to combine the data. Otherwise, a fixed effects model was used. By convention, poor outcome for PD-L1 overexpression was considered when the described HR >1, and would be considered statistically significant if the 95% CI did not overlap 1. Subgroup analysis was further performed to explore the heterogeneity source. Publication bias was investigated by visually assessing the asymmetry of an inverted funnel plot. Moreover, Begg's and Egger's tests were conducted to quantitatively support the publication bias. All analyses were performed using STATA 12.0(STATA Corporation, College Station, TX). A *P*-value <.05 was considered statistically significant.

## 3 Results

### 3.1 Study characteristics

**Figure 1:**
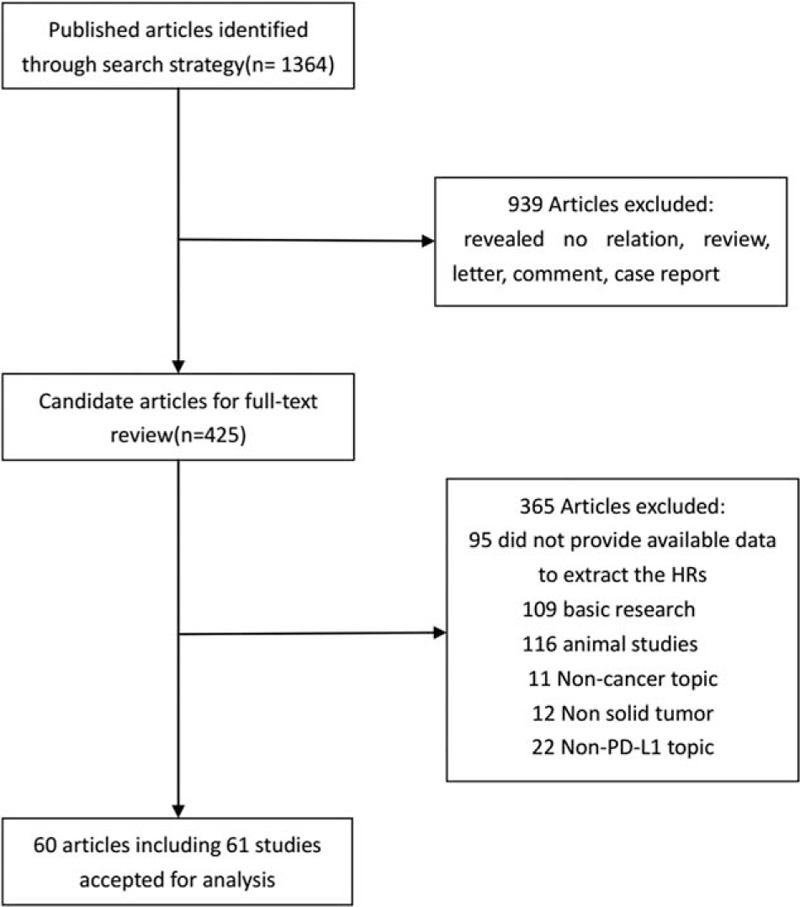
Flow diagram of the study inclusion.

According to the criteria mentioned previously, a total of 1364 articles on PD-L1 in tumors were identified from a primary literature search in PubMed, Embase, Google Scholar, and Cochrane Library. However, 939 irrelevant abstracts were excluded. After manually screening the full-text, we excluded 365 articles, such as basic research, animal studies, noncancer subject, nonsolid tumor, non-PD-L1 topic, or if the data of HRs or OS were unavailable. The remaining 60 articles contained 61 studies, because 1 article included 2 independent cohort studies.^[11]^ Thus, 61 studies were included in this meta-analysis (Fig. 1).

The characteristics of the included studies are listed in Table 1  . The total number of patients in all studies was 10,310, ranging from 23 to 1420 patients. The median follow-up time ranged from 0.75 to 10 years. The category of tumors included esophageal carcinoma (3 studies), gastric cancer (6 studies), oral squamous cell carcinoma (4 studies), hepatocellular carcinoma (HCC) (4 studies), nonsmall cell lung cancer (NSCLC) (13 studies), urothelial cancer (4 studies), breast cancer (4 studies), renal carcinoma (7 studies), melanoma (5 studies), colorectal cancer (4 studies), ovarian cancer (1 study), cervical carcinoma (1 study), pancreatic cancer (1 study),thymic epithelial tumor (1 study), and malignant pleural mesothelioma (2 studies). Thirty-six studies (60%) were reported on Asians, and 24 (40%) studies on Caucasians. The endpoints OS and DFS/PFS were discussed in 60 and 23 studies, respectively. The cut-off values of PD-L1 varied across different studies. The HR estimations for 20 studies were directly reported, whereas others were calculated from the Kaplan–Meier curves given by the articles. Only 48.3% studies performed blinded reading in evaluating PD-L1.

**Table 1:**
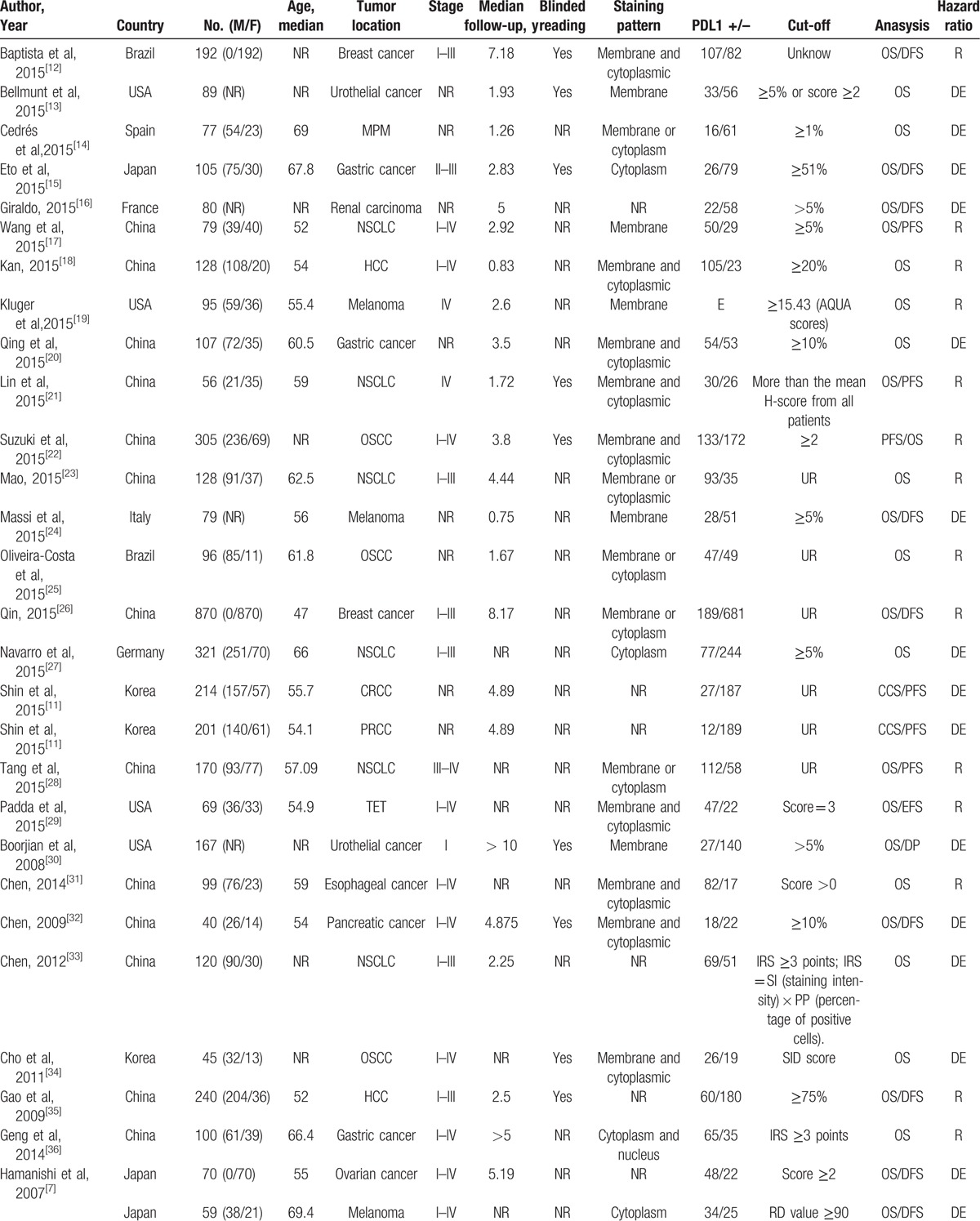
Main characteristics of all studies included in the meta-analysis.

**Table 1 (Continued):**
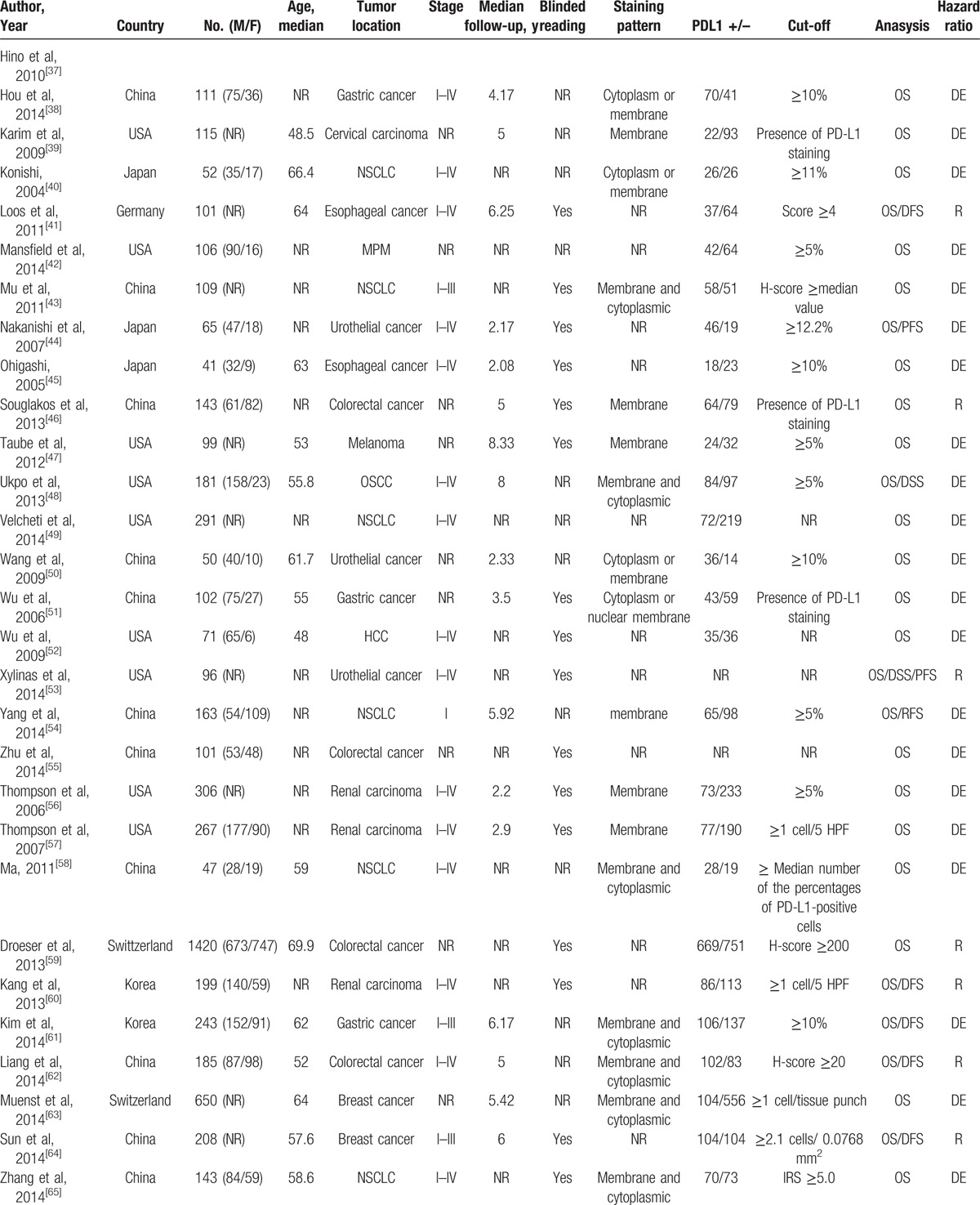
Main characteristics of all studies included in the meta-analysis.

**Table 1 (Continued):**
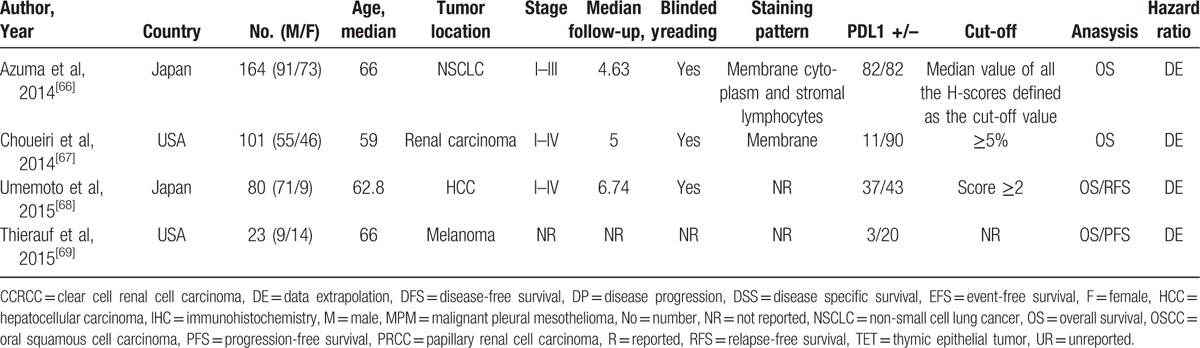
Main characteristics of all studies included in the meta-analysis.

### 3.2 Meta-analysis

**Figure 2:**
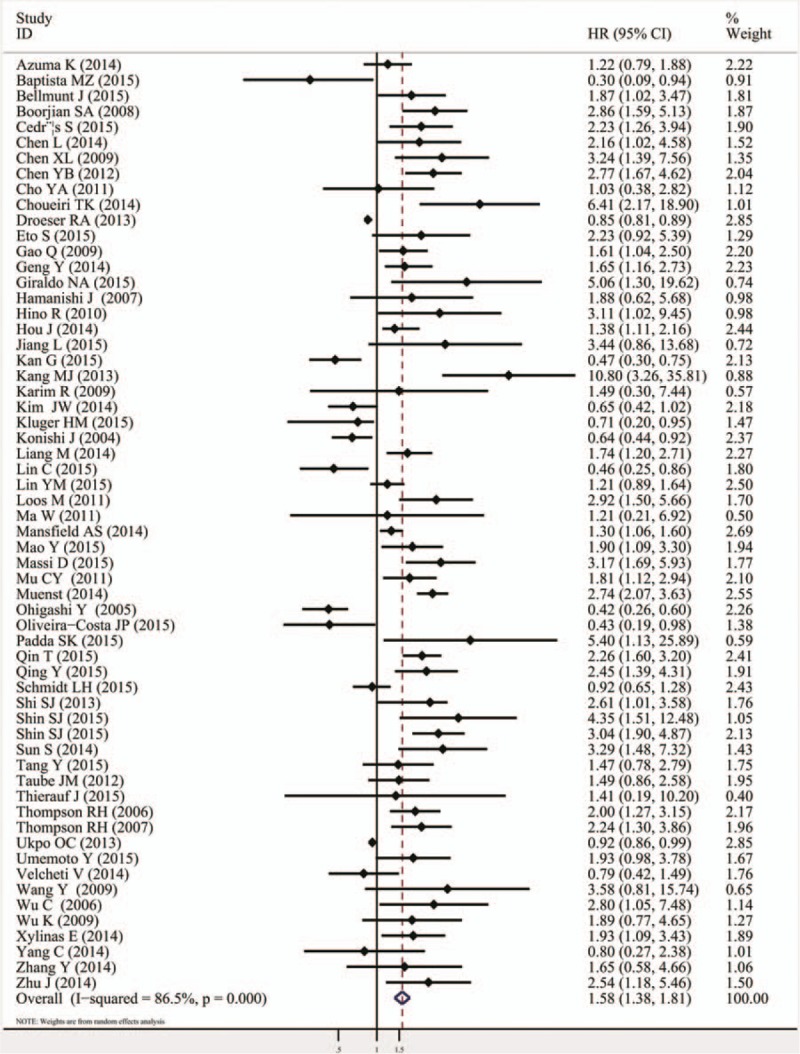
Meta-analysis (forest plot) of 60 PD-L1 evaluation studies included in the overall survival. PD-L1 = programmed death-ligand 1.

**Figure 3:**
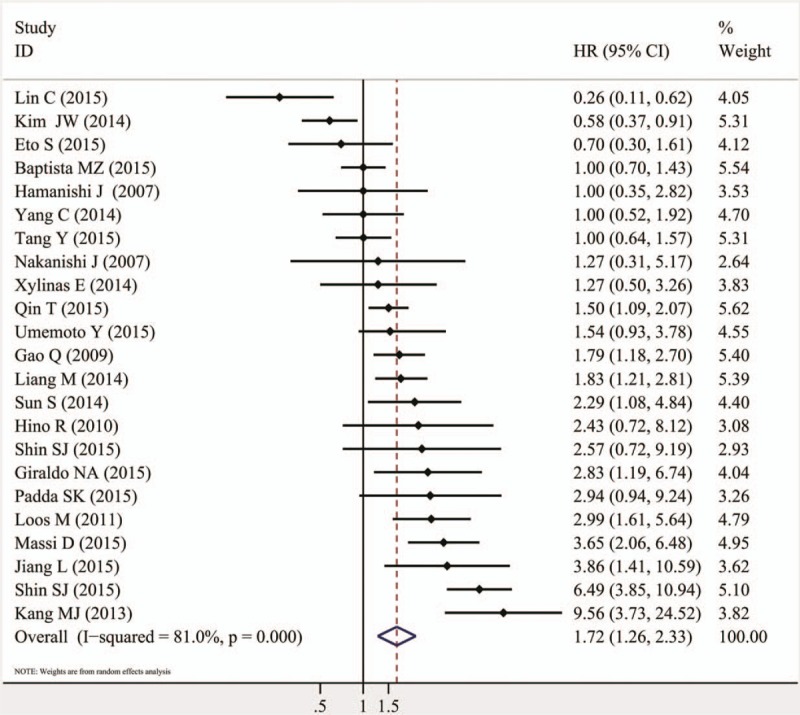
Meta-analysis (forest plot) of 23 PD-L1 evaluation studies included in the PFS/DFS. DFS = disease-free survival, PD-L1 = programmed death-ligand 1, PFS = progression-free survival.

The combined HR of 60 included studies, which included 10,310 cancer patients, showed that the PD-L1 overexpression was associated with poor OS (HR = 1.58, 95% CI = 1.38–1.81, *P* <.000). Furthermore, significant heterogeneity was noted among the studies (*I*^*2*^ = 86.5%, *P* <.000), as shown in Fig. 2 and Table 2. As for PFS/DFS, the pooled HR of 23 eligible studies that included 3821 cancer patients was 1.72 (95% CI = 1.26–2.33, *P* = .001), which suggested that PD-L1 overexpression was a poor prognosis indicator for multiple solid cancers, and significant heterogeneity was found across the studies (*I*^*2*^ = 81%, *P* <.000), as shown in Fig. 3 and Table 2.

**Table 2:**
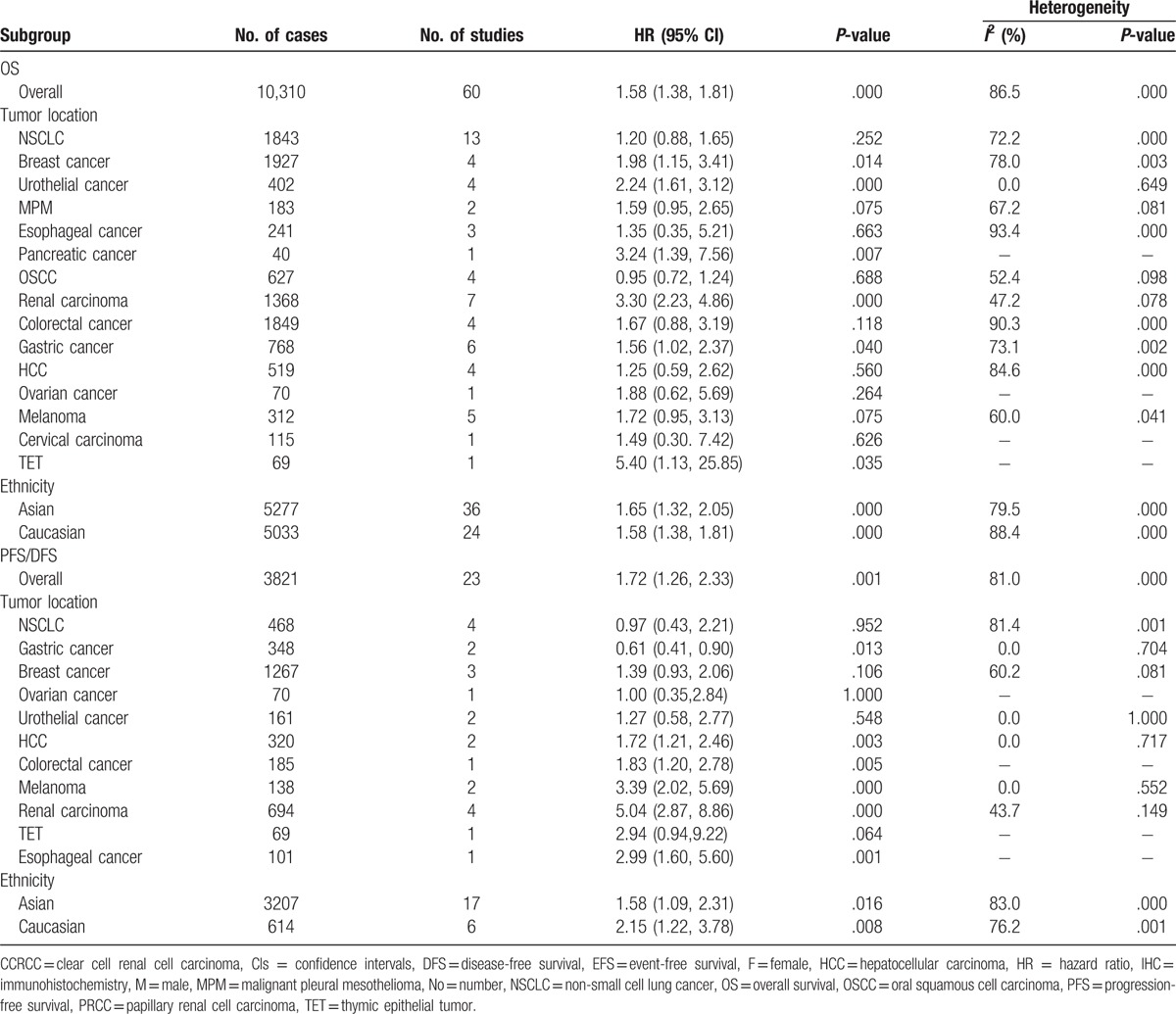
Meta-analysis: HRs value of OS and PFS in overall and subgroups of solid tumor.

### 3.3 Subgroup analysis

**Figure 4:**
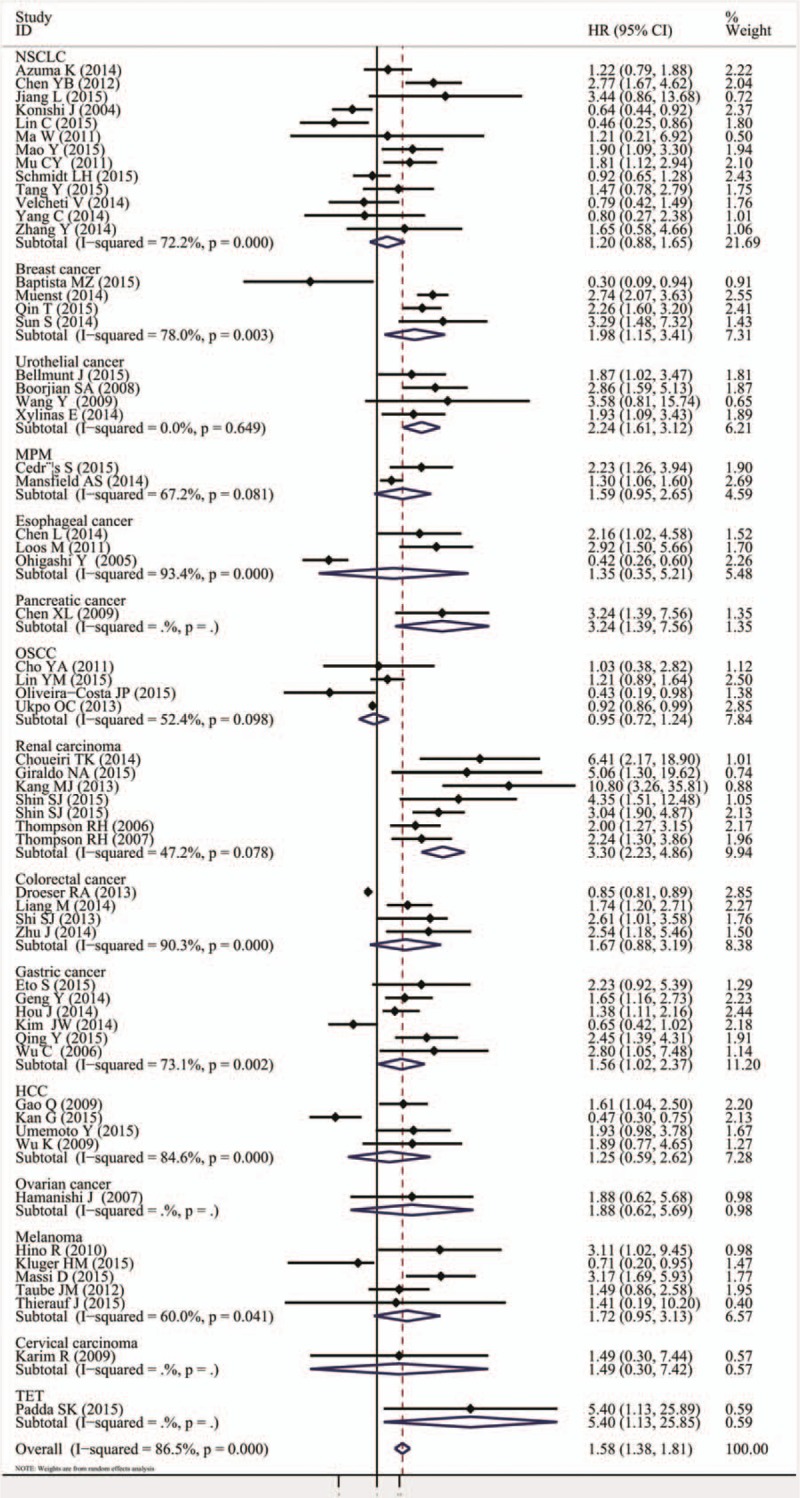
Forrest plots of the subgroup analysis (tumor type) for the effect of PD-L1 overexpression on OS in patients with solid tumors. OS = overall survival, PD-L1 = programmed death-ligand 1.

**Figure 5:**
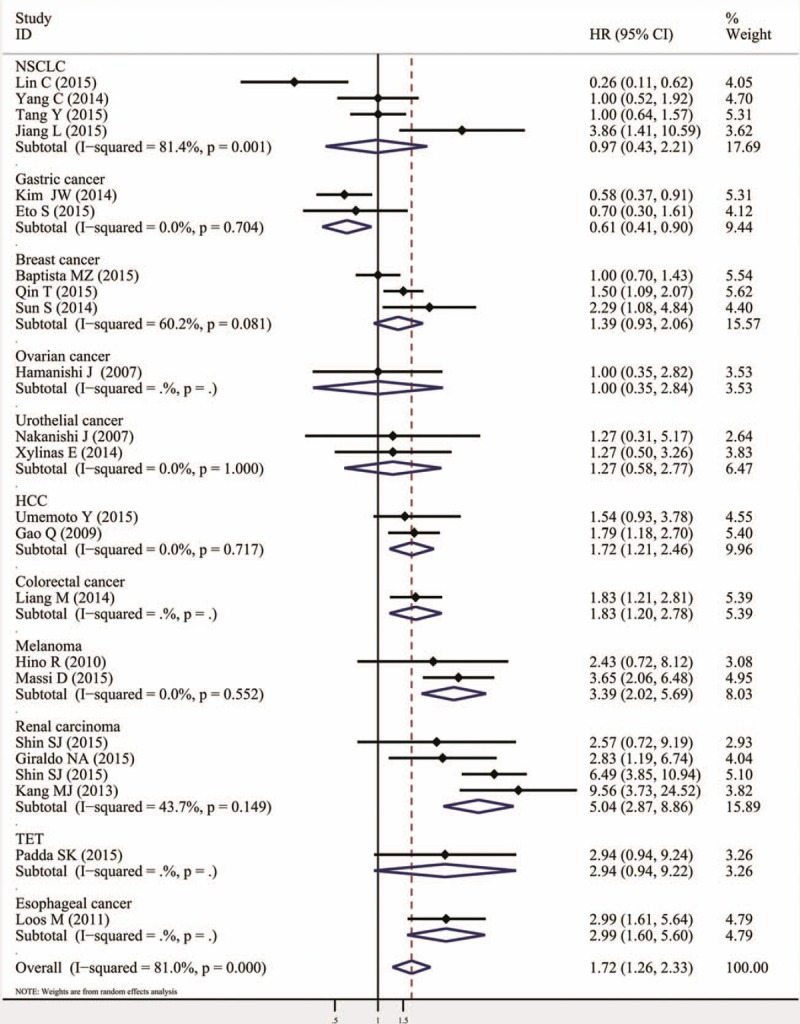
Forrest plots of the subgroup analysis (tumor type) for the effect of PD-L1 overexpression on DFS/PFS in patients with solid tumors. DFS = disease-free survival, PD-L1 = programmed death-ligand 1, PFS = progression-free survival.

The analysis by tumor type is presented in Table 2. The numbers of studies ≥2 showed the association of PD-L1 overexpression with poor OS in breast (HR = 1.98, 95% CI = 1.15–3.41, *P* = .014), urothelial (HR = 2.24, 95% CI = 1.61–3.12, *P* <.000), renal (HR = 3.30, 95% CI = 2.23–4.86, *P* <.000), and gastric cancers (HR = 1.56, 95% CI = 1.02–2.37, *P* = .040). No significant correlation between PD-L1 and OS was found in the other subgroup analyses, as shown in Fig. 4 and Table 2. The DFS/PFS subgroup analysis showed that the PD-L1 overexpression was associated with shorter DFS/PFS in HCC (HR = 1.72, 95% CI = 1.21–2.46, *P* = .003), melanoma (HR = 3.39, 95% CI = 2.02–5.69, *P* <.000), and renal carcinoma, (HR = 5.04, 95% CI = 2.87–8.86, *P* <.000; Fig. 5, Table 2).

**Figure 6:**
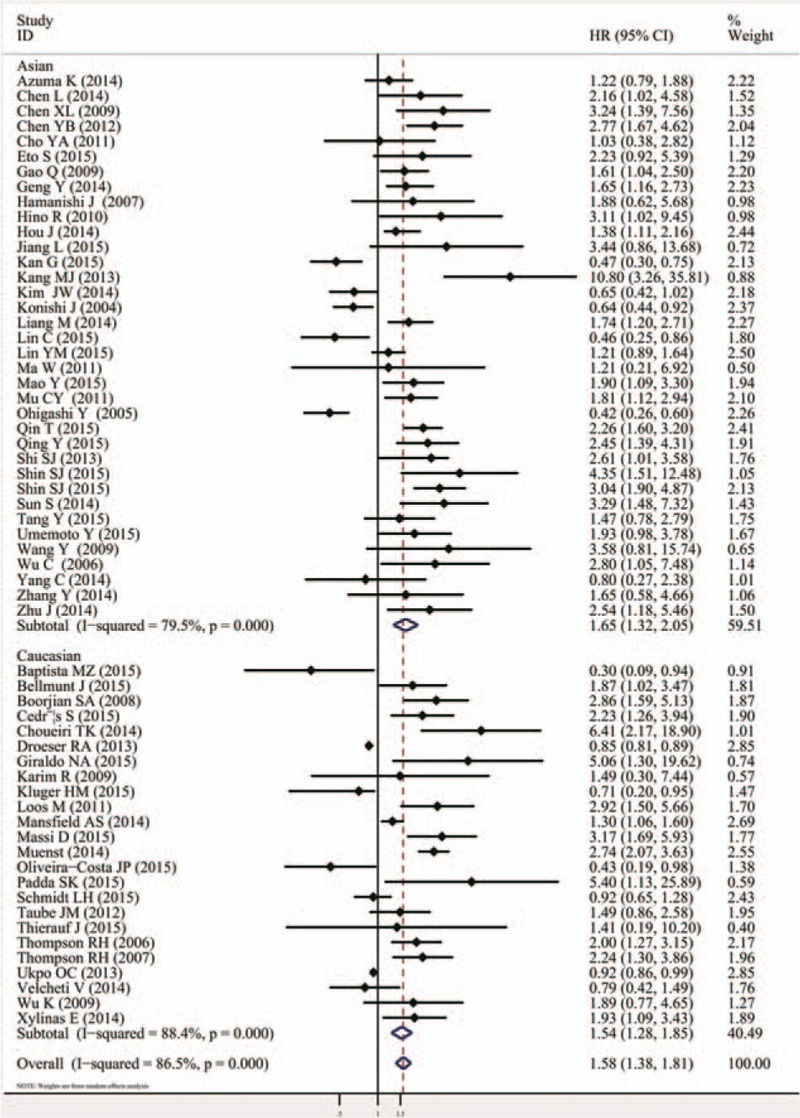
Forrest plots of the subgroup analysis (ethnicity) for the effect of PD-L1 overexpression on OS in patients with solid tumors. OS = overall survival, PD-L1 = programmed death-ligand 1.

**Figure 7:**
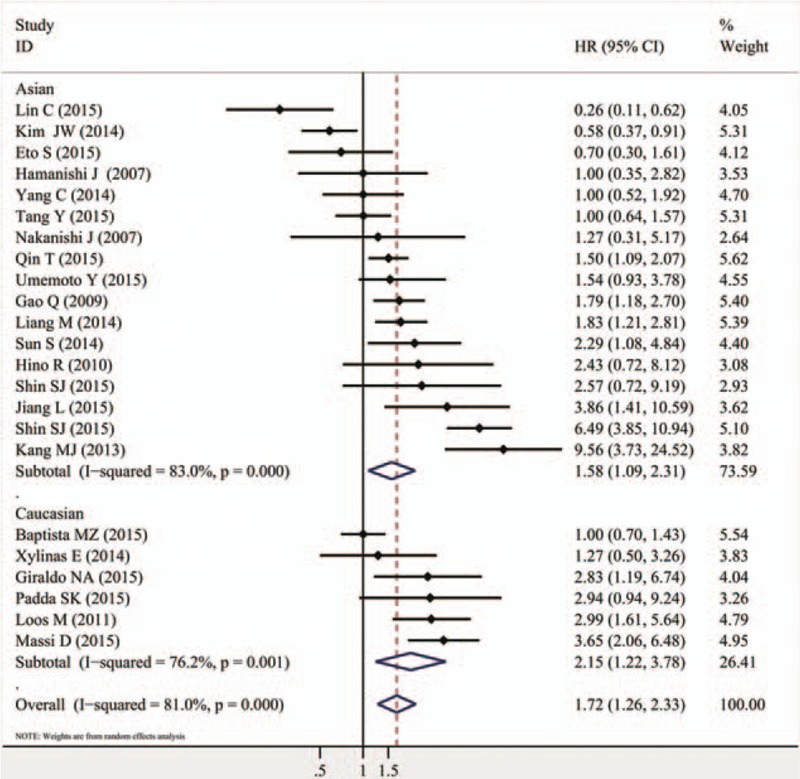
Forrest plots of the subgroup analysis (ethnicity) for the effect of PD-L1 overexpression on DFS/PFS in patients with solid tumors. DFS = disease-free survival, PD-L1 = programmed death-ligand 1, PFS = progression-free survival.

The other subgroup analysis on ethnicity indicated that the PD-L1 overexpression was associated with poor OS in either Asian (HR = 1.65, 95% CI = 1.32–2.05, *P* <.000; *I*^*2*^ = 79.5%, *P* <.000) or Caucasian patients (HR = 1.58, 95% CI = 1.38–1.81, *P* <.000; *I*^*2*^ = 88.4%, *P* <.000; Fig. 6, Table 2). Moreover, the same findings were observed for the DFS/PFS in Asian (HR = 1.58, 95% CI = 1.09–2.31, *P* = .016; *I*^*2*^ = 83.0%, *P* <.000) and Caucasian patients (HR = 2.15, 95% CI = 1.22–3.78, *P* = .008; *I*^*2*^ = 76.2%, *P* = .001; Fig. 7, Table 2).

### 3.4 Publication bias and sensitivity analysis

**Figure 8:**
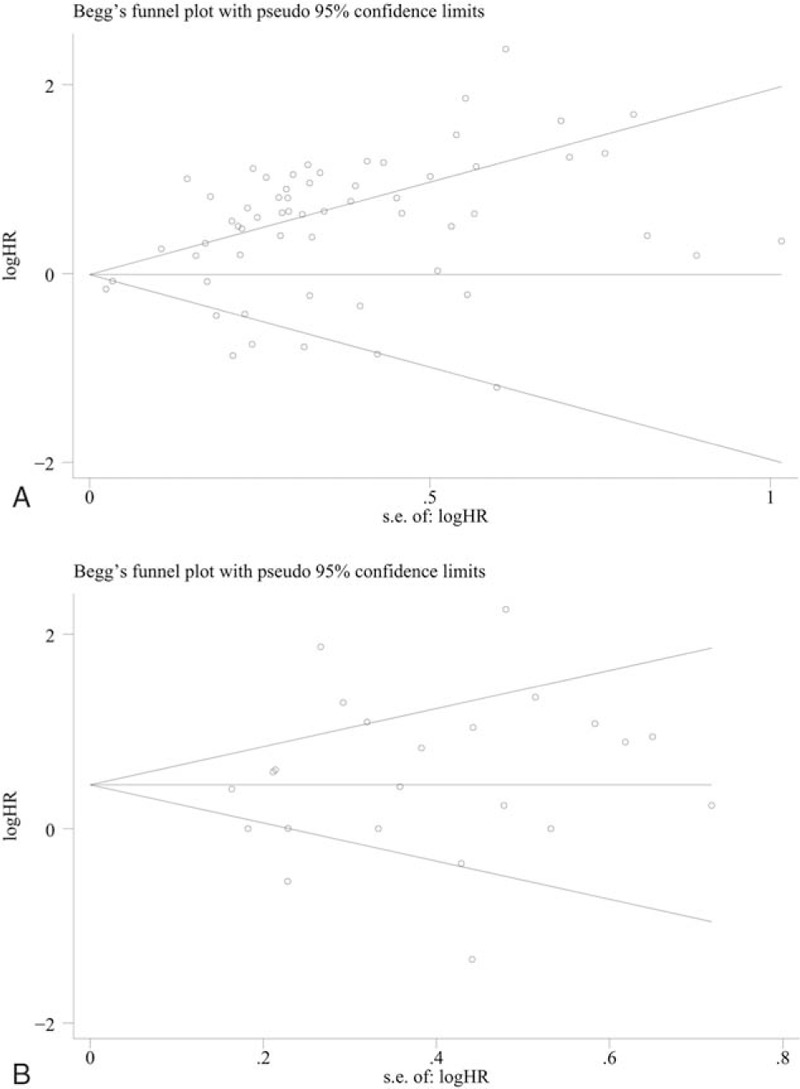
Funnel plots of the publication bias summary for the corresponding meta-analysis in (A) OS, (B) DFS, or PFS. DFS = disease-free survival, OS = overall survival, PFS = progression-free survival.

We chose Begg's funnel plot and Egger's test to detect the publication bias of our meta-analysis. A total of 23 studies evaluating the DFS/PFS of patients with multiple tumors yielded a Begg's and Egger's test scores of *P* = .460 and *P* = .379, respectively. At the same time, no publication biases were observed based on the funnel plot (Fig. 8B). However, the evaluation of the PD-L1 overexpression on the OS of patients in 60 studies yielded publication biases (Begg's test, *P* = .549 and Egger's test, *P* <.001) (Fig. 8A). Sensitivity analysis demonstrated that deleting any single study did not significantly affect the pooled HRs for OS and DFS/PFS (Supplementary Figs. 1 and 2).

## 4 Discussion

PD-1 and its ligands, PD-L1 and PD-L2 expressed within the tumor microenviroment, can modulate the balance between T-cell activation, tolerance, and immunopathology during long-term antigen exposure.^[70,71]^ To evade from the immune system's monitoring, tumor cells in tumor microenvironment can upregulate the expression of PD-L1 through a variety of mechanisms, and bind themselves with negative immune checkpoint PD-1 on the surface of T cells, thereby inhibiting T cells function, losing its killing effect on tumor cells.^[71]^

The correlations between PD-L1 expressions and different tumors have been studied by numerous researches. However, the results were inconsistent and conflicting. To arrive at a reasonable conclusion, we performed this meta-analysis and aimed to evaluate the relationship between PD-L1 overexpression and the prognosis of solid tumor patients. Our analysis included 60 studies with 10,310 cases. The results showed that PD-L1 overexpression is a poor prognostic factor in multiple solid tumors with statistical significances for OS (HR = 1.58, 95% CI = 1.38–1.81), and DFS/PFS (HR = 1.72, 95% CI = 1.26–2.33). We conducted subgroup analyses according to different tumor types and ethinicities. For tumor type, the analysis indicated a statistically significant detrimental effect of PD-L1 on the OS in breast, urothelial, renal, and gastric cancers. Furthermore, PD-L1 overexpression was also significantly associated with worse PFS in HCC, melanoma, and renal carcinoma. For ethnicities, the adverse prognostic impact of PD-L1 was retained in patients with different ethnicities. Other studies showed that antibodies blocking PD-1 or PD-L1 are likely to provide a new benchmark for antitumor activity in immunotherapy.^[72,73]^ Several clinical trials, which combine anti-PD-1 mAbs with cancer vaccines (melanoma, prostate cancer, renal cell carcinoma, and angiomyolipoma), antitumor mAbs (lymphoma), or chemotherapies (pancreatic cancer and NSCLC), have been planned or are in progress.^[74]^ Thus, PD-L1 may be used as a prognostic marker and therapeutic target for those cancers, which provides impetus for the further investigation of its functions.

The robustness of the results in our meta-analysis was confirmed by a sensitivity analysis, which did not significantly alter the pooled effect size for OS and DFS/PFS. Furthermore, HRs extracted from Kaplan–Meier curves seemed to be less reliable than those reported directly. However, we did not discover any obvious primary deviation in the publications when comparing our extracted OS HRs and 95% CIs with the outcomes of the published studies. Nevertheless, this meta-analysis still has many deficiencies. First, only published studies and studies in English were included. We may have missed unpublished and non-English studies which are unsuitable for meta-analysis, meanwhile there might be selection bias. Second, high heterogeneity was observed among studies, which will influence the analysis, interpretation, and conclusions of this study. To minimize the effect of the heterogeneity on OS and DFS/PFS, we used a random effect model. Nevertheless, the heterogeneity within and between studies only disappeared in urothelial cancer, melanoma, and renal carcinoma subgroups, we could not determine the origin of the heterogeneity. Third, no consistent standard for cut-off values were found in our included studies, which can cause clinical and statistical heterogeneity. Finally, the publication bias between the relationship between PD-L1 overexpression and OS has been shown on Egger tests and funnel plots, and we found evidence of the publication biases across the included studies. However, our finding that the corrected pooled effect size remained statistically significant after using the “Trim and Fill” method to adjust for the effect of potential publication bias on the results of the meta-analyses. Thus, the reliability of our results could be confirmed. In addition, studies with different follow-up times can also result in bias.

## 5 Conclusions

In conclusion, we demonstrated that PD-L1 overexpression is significantly associated with poor OS and DFS/ PFS, especially in renal carcinoma and urothelial cancer. PD-L1 overexpression may predict poor prognosis in different cancers. Furthermore, the PD-L1 target can become an effective anti-cancer therapy as well.

## Acknowledgments

Authors thank all the authors whose publications were included in our meta-analysis.

## Supplementary Material

Supplemental Digital Content
