# Supplementary material for: Prognostic significance of PD-L1 in solid tumor: An updated meta-analysis
Source: Medicine (Baltimore). 2017 May 5;96(18):e6369. doi: 10.1097/MD.0000000000006369 (PMC5419898; doi:10.1097/MD.0000000000006369)

**Supplemental Fig 1. Result of sensitivity analysis of PD-L1 overexpression and OS in patients with solid tumor**

**
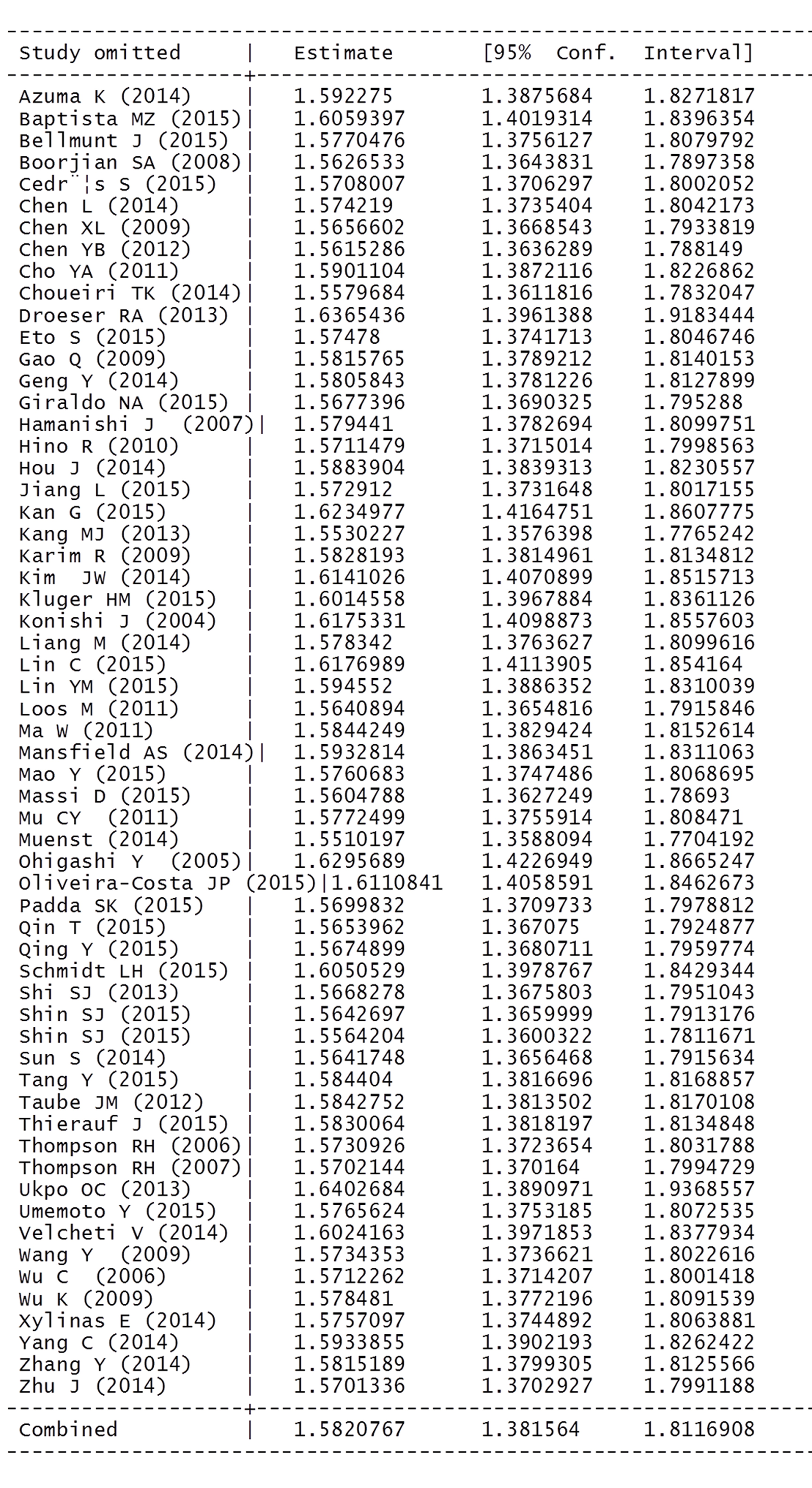
**

**Supplemental Fig 2.** **Result of sensitivity analysis of PD-L1 overexpression and DFS/PFS in patients with solid tumor**


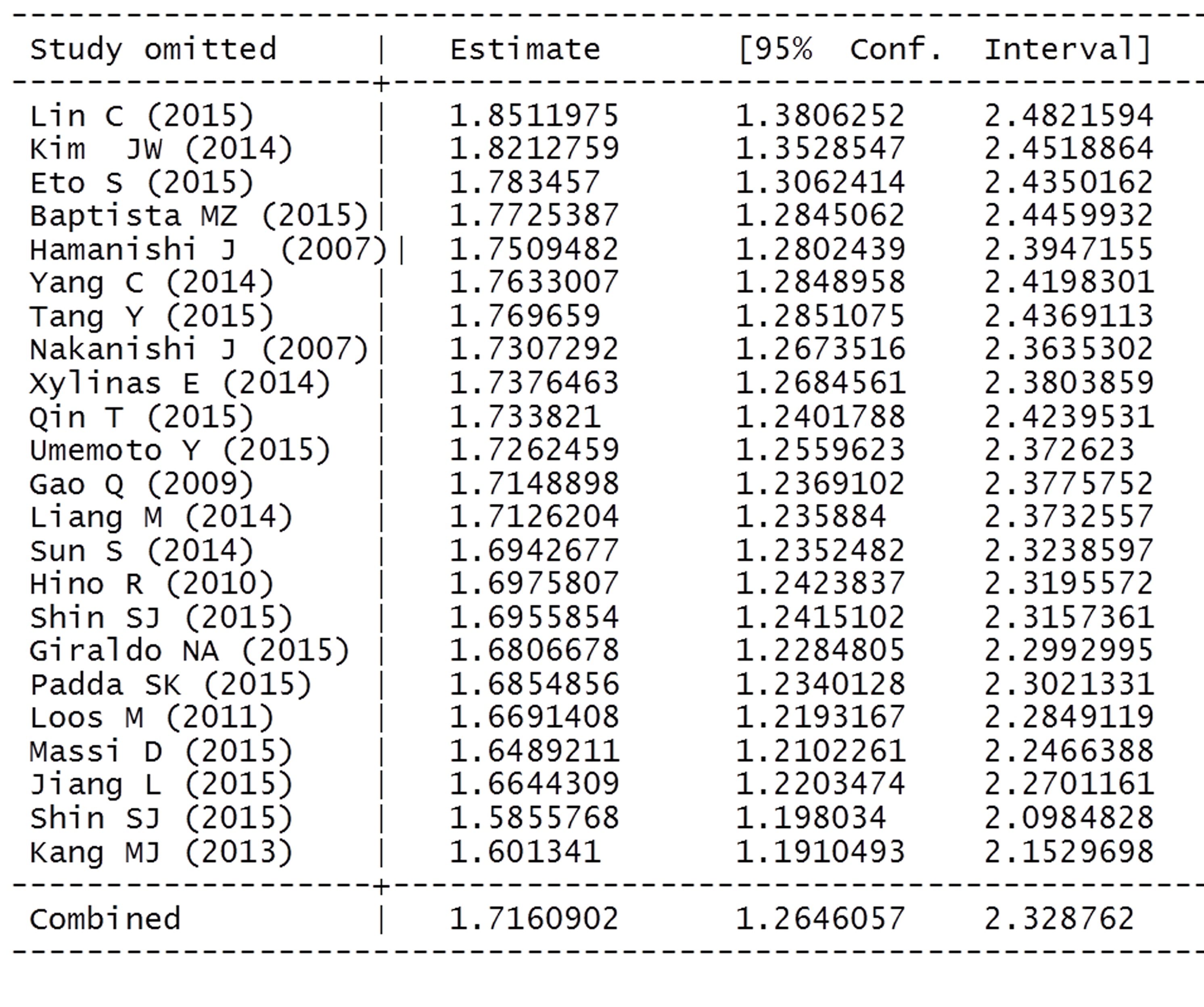

Supplement: Supplemental Digital Content [file medi-96-e6369-s001.doc]
